# Vigilance for Medical Products of Human Origin—Progress on the Notify Library’s Global Effort to Share Information and Learning

**DOI:** 10.1097/TP.0000000000003589

**Published:** 2021-08-19

**Authors:** Evangelia Petrisli, Claudia Carella, Aurora Navarro, Deirdre Fehily, Douglas Michael Strong, Massimo Cardillo

**Affiliations:** 1 Operative Unit of Microbiology and Virology, Department of Specialized, Experimental, and Diagnostic Medicine, IRCCS St Orsola-Malpighi Polyclinic, University of Bologna, Bologna, Italy.; 2 National Transplant Centre, Istituto Superiore di Sanità, Rome, Italy.; 3 Department of Biovigilance, Catalan Transplant Organization, Health Department, Generalitat of Catalonia, Barcelona, Spain.; 4 Directorate General for Health and Food Safety, European Commission, Brussels, Belgium (seconded from the Italian National Transplant Centre, Rome, Italy).; 5 Department of Orthopaedics and Sports Medicine, University of Washington School of Medicine, Seattle, WA.

## Abstract

World Health Assembly Resolution 63.22 mandated World Health Organization to facilitate Member State access to appropriate information on medical products of human origin (MPHO), including collecting data on serious adverse events and reactions. To meet this challenge, the Italian National Transplant Center, with a mandate from World Health Organization, has built and maintained an open-access searchable database of instructive records on disease transmission and other MPHO adverse occurrences.

**Methods.:**

One record in the Notify Library describes a specific type of adverse occurrence in 1 type of MPHO and might be linked with 1 or multiple different references. The record inclusion criteria are that it has been reliably documented in a published article or official vigilance reporting system and that it has instructive value for the fields of transfusion, transplantation, or assisted reproduction. The selection and review of references for publication is performed by international experts who collaborate in 5 topic-specific editorial groups: infection transmission, malignancy transmission, living-donor reactions, process-related incidents, and clinical complications. New relevant references are identified through systematic searches and proactive communication by the experts.

**Results.:**

The Library contains 1733 records, quoting 2632 references. Of the records, 41.8% are related to organs, 20.8% to blood and blood components, 16.5% to hematopoietic progenitor cells, 15.2% to tissues, 4.2% to reproductive tissues and cells, and 1.5% to other MPHO.

**Conclusions.:**

Notify Library is the first open-access, searchable database of systematically identified reports of disease transmission and other adverse occurrences arising from the donation and clinical application of MPHO.

## Abstract 2

Supplemental Digital Content is available in the text.

## INTRODUCTION

Accomplishments in medical practice and research have led to the clinical application of an increasing number and a wider array of medical products of human origin (MPHO) to sustain and improve the quality of life. From donation to the follow-up care of donors and recipients, however, MPHO have a shared exposure to risks—breaches of ethical, legal, and safety standards, for example, the risk of disease transmission with a potential undesirable outcome.^1^ Since 2012, different types of adverse occurrences associated with the clinical use of MPHO are collected in the Notify Library.

The Notify Library (https://www.notifylibrary.org) is an open-access searchable database of adverse occurrences arising from donation of MPHO to their clinical application and follow-up. A single record in the database describes 1 type of adverse occurrence in 1 type of MPHO, highlighting what can be learned from it. It might be linked with 1 or multiple different references and 1 or multiple individual cases of that type for that MPHO. The Library brings together and shares instructive information on reliably documented adverse occurrences to facilitate improvements in safety, quality, and efficacy in clinical application and to provide greater public transparency on the donation and use of all types of MPHO.

The Notify project was launched in response to World Health Assembly Resolution 63.22^2^ that mandated the World Health Organization (WHO) to facilitate Member State access to appropriate information on human cells, tissues, and organs, including data on serious adverse events and reactions. To fulfill this mandate, WHO requested that the Italian National Transplant Centre (CNT) in its role of collaborating center for the vigilance and surveillance of organs, tissues, and cells^3^ build an open-access database where information could be found on all types of adverse occurrences, on all types of MPHO, and on what can be learned from them.

The Library was developed as a global initiative and is now maintained and updated through the voluntary efforts of a large network of international experts including scientists, clinicians, and regulators. During CNT’s first 2 mandates as a WHO collaborating center, the Notify network of experts has met on an annual basis to review progress and define the project strategy. Over the years, >250 professionals have participated in 1 way or another, many providing ongoing routine support on a voluntary basis. Since 2015, the National Transplant Organization of Spain and the regional Catalan Transplant Organization have been cooperating with CNT on some aspects of the project’s development and dissemination. Acknowledging the importance of this project’s goals, important European and International institutions such as the European Commission, the Pan-American Health Organization, and the Spanish authorities have hosted meetings of the Notify project experts.

The scope of the Notify Library covers blood and blood components, organs, tissues, and cells used in transfusion, transplantation, or in medically assisted reproduction, and other MPHO addressing adverse occurrences in recipients, living donors, and children born from in vitro fertilization.^4^ The scope reflects a recognition that organs, blood components, hematopoietic progenitor cells (HPCs), tissues, reproductive cells, plasma derivatives, human milk, and other products of human origin are part of an exceptional class of medical products. They require a human donor for their procurement and are applied to other human beings for therapeutic purposes, implying particular risks and ethical concerns. MPHO donation and clinical use support key pillars of health services worldwide, and their management requires achieving sufficiency and ensuring ethical practice, safety, and effectiveness. The global consensus on the donation and management of blood and blood components^2^ as well as the WHO principles on human cell, tissue, and organ transplantation^5^ define the key principles to be applied. Because of the exceptional nature of the MPHO source and the often complex activities performed before their use (processing, storage, preservation, distribution, and application), well-defined rules and procedures must be followed to ensure quality and safety of the whole process. Vigilance, surveillance, and traceability are essential elements of the quality management system, completing the continuous process improvement cycle. The Notify Library is a key tool that supports sharing of vigilance information across international borders and between professional sectors working in the MPHO field. The network is the first of its kind addressing vigilance and surveillance across this broad scope. As the use of this resource grows, lessons learned from adverse outcomes in 1 place, or 1 clinical specialty, can provide invaluable input to improving the safety and effectiveness in very different geographical and clinical realities.

This article aims to describe the achievements of the project to date, discuss some of its key strengths and challenges, and point to a number of prioritized objectives that it intends to take forward in the future.

## BUILDING AND UPDATING THE LIBRARY

### Definition of a Notify Record and Inclusion Criteria

An important early step was to clearly define the concept of a single “record” in the Library. A Notify record describes a specific type of adverse occurrence associated with a specific type of MPHO. A record is linked to 1 or more reference sources and may describe 1 or multiple individual cases of the occurrence.

A case is suitable for inclusion in a record of the Notify Library when it meets the following inclusion criteria:

- It describes an adverse occurrence that *has caused harm* to a donor or a recipient of an MPHO or to a fetus or embryo created through gamete or embryo donation or an adverse occurrence that *has presented a risk of harm*.
- It is *reliably documented* in the scientific, clinical, or legal literature or in a formal professional or regulatory vigilance program.
- It has *instructive value*.

Cases are considered to have instructive value when they describe in detail common occurrences, as well as occurrences that were detected through unusual signs or symptoms in the recipient or donor or novel approaches to investigation of the cause. Uncommon or unexpected causes or errors, unforeseen levels of severity, or new mitigation or corrective actions also make individual cases instructive. These cases often help to improve the estimation of risk for donation or clinical application or point to a need to implement changes in routine procedures to avoid recurrence. Cases that did not cause harm but had a significant potential to do so can also constitute informative records in the Library, and those where a known or unknown risk did not result in harm are also highly instructive. The Library is not a vigilance reporting system that aims to gather all cases of adverse occurrences. It is, rather, a compendium of records describing what can be learned from different types of occurrences and risks that are well documented.

### Search Methodology

In the early stages, experts used reviews or their own reference libraries and vigilance system reports to identify the information to be inserted. Initially, over 1700 published references describing adverse occurrences were identified in preparation for summary reports. The data collection exercise resulted in a compendium of references, which were entered in an Endnote library and subsequently uploaded to the Notify database once it was established. During the first global meeting of >100 experts held in Bologna, Italy, in 2011, the cases were recategorized by adverse occurrence type and reviewed by editorial groups comprising experts in each of the following specific areas: infection transmission, malignancy transmission, genetic transmission, living-donor reactions, and process-related errors or incidents. A summary of that meeting was published later that year.^6^ Initially, blood-related cases were not included but have been added a few years later following a meeting in Barcelona, Spain.^7^

**FIGURE 1.**
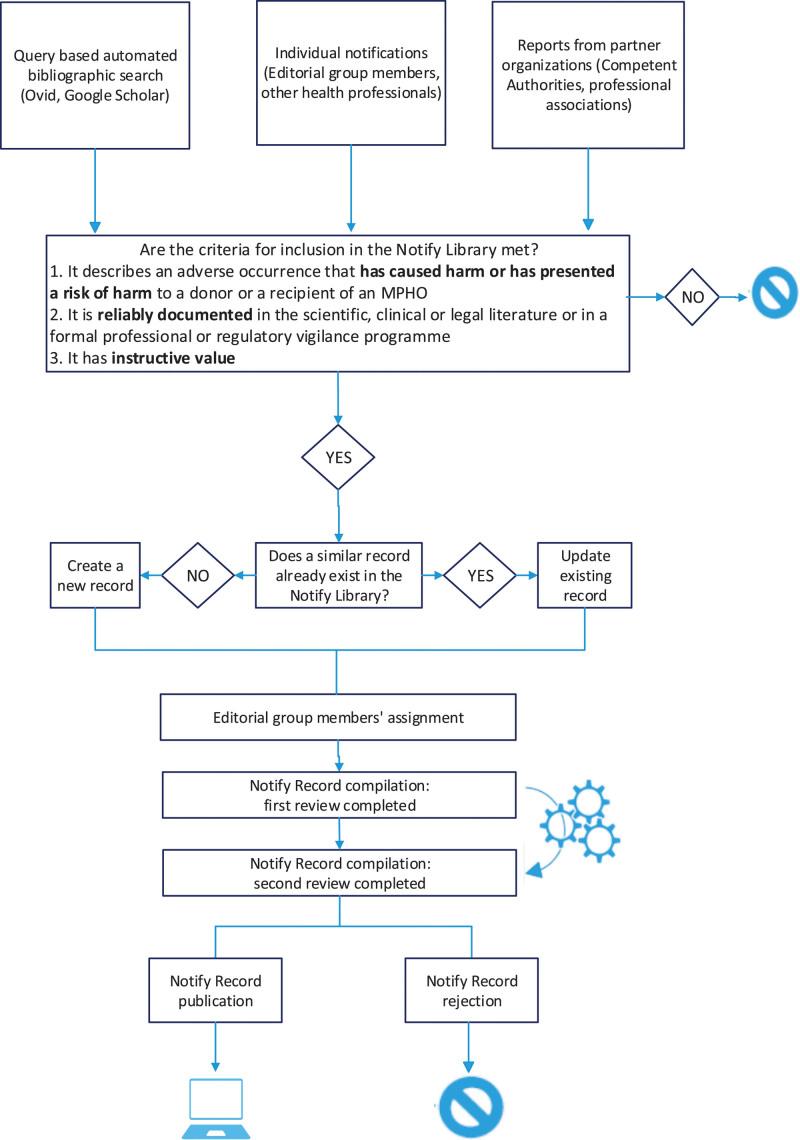
Current search methodology and editorial group case evaluation process. MPHO, medical products of human origin.

The process has become significantly more structured over time. The current search methodology is included in Figure 1. References are identified primarily by literature review, through a query-based automated search on Ovid and Google Scholar platforms, individual notifications from editorial group members or other health professionals, weekly updates, newsletters, and publication alerts from the major journals in the fields of transfusion and organ, tissue, and cell transplantation. Regulatory or professional vigilance reports (gray literature) from collaborating organizations are also evaluated for identification of cases for inclusion in the Notify Library. The latter represents an important source of cases with instructive values that are usually not published in the medical literature because their documentation is limited to internal quality system records or regulatory reporting systems.

### Editorial Group Case Evaluation

The selection and review of cases and publications for inclusion in the Library is carried out by international experts of the transfusion, transplantation, medically assisted reproduction communities, and other health professionals (Figure 1). They collaborate in 5 topic-specific editorial groups: *infection transmission*, *malignancy transmission*, *living-donor reactions*, *process-related incidents*, and (other) *clinical complications*. Editorial groups are composed to ensure that they have expertise from all the MPHO specialties and each group has a chair and cochair. They perform a structured analysis of the reported cases according to the agreed standard operating procedures and technical guidelines that are periodically reviewed. A formal policy of having 2 reviewers per record has been established in all editorial groups to avoid bias and improve robustness.

Each record analysis describes latency, estimated frequency (eg, synopsis of the Council of Europe risk assessment and recommendations for the various tumor types), signs, symptoms, or how the occurrence was detected and the methods used to assess imputability (ie, the probability that the adverse outcome in the donor or in the recipient was associated with the donation, process, or clinical application of that specific MPHO). Based on the information that is available in the cited reference or vigilance and surveillance program report, the imputability is subsequently categorized as proven, probable, possible, unlikely, or excluded, according to the criteria provided in Table 1.

**TABLE 1.** Imputability table used for the assessment of cause and effect for each Notify record

**Imputability Grade**	**Criteria for infectious and malignant transmissions adapted from DTAC** ^8^	**Adapted from EUSTITE—SOHO V&S** ^9^ **and proposed standard definitions for surveillance of noninfectious adverse transfusion reactions** ^10^	**Adapted from EUSTITE—SOHO V&S in assisted reproductive tecnologies** ^9^
Not assessable	Insufficient data for imputability assessment	Insufficient data for imputability assessment	Insufficient data for imputability assessment
Excluded	Suspected transmission and fulfillment of at least one of the following conditions:	Conclusive evidence beyond a reasonable doubt that the adverse occurrence can be attributed to causes other than the transfusion of blood components or transplantation of tissues/cells	Conclusive evidence beyond reasonable doubt for attributing to alternative causes than the ART process
	- Clear evidence of an alternative cause.- The appropriate diagnostic tests performed have failed to document infection by the same pathogen in any recipient from the same donor.		
	Laboratory evidence that the recipient was infected with the same pathogen or had a tumor before the application of organs, tissues, or cells.		
Possible	Suspected transmission and:- Laboratory evidence of the pathogen or tumor in a single recipient, *or*	The evidence is indeterminate for attributing the adverse occurrence either to the quality/safety of tissues/cells/blood components (for recipients), to the donation process (for donors), or to alternative causes	Evidence is indeterminate
	Suspected transmission and: - Laboratory evidence of the pathogen or tumor in a single recipient or - Data suggest a transmission but are insufficient to confirm it.		
Likely/probable	The following 2 conditions are met: - Suspected transmission. - Laboratory evidence of the pathogen or the tumor in a recipient.	The evidence is clearly in favor of attributing the adverse occurrence to the quality/safety of tissues/cells/blood components (for recipients) or to the donation process (for donors)	The evidence is in favor of attributing to the ART process
	And it meets at least one of the following conditions:- Laboratory evidence of the same pathogen or tumor in other recipients.- Laboratory evidence of the same pathogen or tumor in the donor.		
	If there is pretransplant laboratory evidence, such evidence must indicate that the same recipient was negative for the pathogen involved before transplantation.		
Definite/certain; proven	All the following conditions are met: - Suspected transmission.- Laboratory evidence of the pathogen or the tumor in a recipient.- Laboratory evidence of the same pathogen or tumor in other recipients (if multiple recipients).- Laboratory evidence of the same pathogen or tumor in the donor.- If there is pretransplant laboratory evidence, then it should be noted that the same recipient was negative for the pathogen before transplantation.	The evidence is conclusive beyond reasonable doubt for attributing the adverse occurrence to the quality/safety of tissues/cells/blood components (for recipients) or to the donation process (for donors)	Conclusive evidence beyond reasonable doubt for attributing to the ART process

ART, assisted reproduction technologies; DTAC, Disease Transmission Advisory Committee; EUSTITE, European Union Standards and Training for the Inspection of Tissue Establishments Project (GA 2005204); SOHO V&S, Vigilance and Surveillance of Substances of Human Origin Project (GA 20091110).

For the last 3 years, editors were invited to also add “expert comments” to individual records, thus providing an added instructive value to the Library’s content. Table 2 shows the basic dataset for each Notify record.

**TABLE 2.** Parameters included in each Notify record with an illustrative example (Notify record ID: 1861)

Adverse occurrence description	Case report: donor-derived metastatic melanoma (kidney transplant)
Adverse occurrence type	Harm to recipient => malignancy => skin => melanoma
MPHO type	Organ => kidney
Time to detection	6 mo after transplantation of the kidney
Alerting signals, symptoms, evidence of occurrence	Severe fatigue and shortness of breath in kidney recipient with mild tenderness to palpation in the right lower quadrant along the allograft site. A CT scan noted innumerable punctate nodules in the lungs, a 1–3 cm hypoattenuating lesion in the right hepatic dome, and scattered nonenlarged para-aortic lymph nodes. A CT-guided percutaneous biopsy of a left lower lobe pleural-based nodule was performed; pathology revealed melanoma with a BRAF-V600E mutation. After allograft nephrectomy, findings demonstrated extensive tumor throughout the kidney allograft. Pathology consistent with melanoma with lymphovascular invasion. There was a liver recipient from the same donor that was also diagnosed with donor-derived metastatic melanoma, but there is no further information on that case.
Estimated frequency	Melanoma: most recent risk assessment for melanoma (Council of Europe, 2018): donors with active melanoma represent an unacceptable risk for organ donation. Donors with a history of treated melanoma are generally considered to represent a high transmission risk. Opinions vary. The SaBTO/United Kingdom states that a superficial spreading melanoma with tumor thickness <1.5 mm and with curative surgery and cancer-free interval of >5 y is associated with a low transmission risk, although this conclusion is based on a small number of cases. UNOS/DTAC considers all patients with a history of melanoma to represent a high risk for transmission.
Demonstration of imputability or root cause	HLA typing of the tumor cells matched that of the donor, indicating a diagnosis of donor-derived metastatic melanoma. A female liver recipient from the same donor (only other recipient) was also diagnosed with donor-derived metastatic melanoma, determined by identification of male karyotype in melanoma cells.
Imputability grade	Definite/certain/proven
Keywords	malignancy, case report, kidney, kidney transplant, liver, liver transplant, melanoma, deceased donor
Reference	11
Expert comment for publication	This is an interesting case report of a kidney recipient who was himself a previous donor who developed chronic glomerulonephritis. The donor for this patient had no evidence of malignancy, and this case report emphasizes the need for clinicians to maintain a high index of suspicion for donor-derived disease in patients who have atypical presentations after a kidney transplant. Although melanoma transmission is typically associated with high mortality, this report highlights successful management with the use of targeted therapies following transplant nephrectomy. The tumor was shown to have a BRAF V600E mutation, and the BRAF inhibitor dabrafenib was used along with the MEK inhibitor trametinib, leading to clinical response but noncompliance due to side effects. For that reason, nivolumab was initiated with marked clinical response. The authors provide details regarding the use of these drugs in patients with compromised renal function. Context is important, and it should be remembered that the use of checkpoint inhibitors is likely to cause immune stimulation, increasing the likelihood of organ rejection.

CT, computerized tomography; DTAC, Disease Transmission Advisory Committee; MPHO, medical products of human origin; SaBTO, Advisory Committee on Safety of Blood, Tissues and Organs; UNOS, United Network for Organ Sharing.

### Notify Library Structure

To allow structured database searches, each Notify record is classified according to a taxonomy divided into 2 parts:

1. Adverse occurrence types (harm to a recipient, harm to a donor, harm to a fetus or offspring, risk of harm).
2. MPHO types (organs, blood components, HPCs, tissues, reproductive cells, plasma derivates, human milk, and other products of human origin).

Both are further organized in a tree-like structure, with parent-child relationships between the different levels of hierarchy as shown in Supplement Annex I (SDC, http://links.lww.com/TP/C89). New subcategories are added as the database is continuously updated with new adverse occurrence types or new MPHO types. Finally, a list of keywords for each record is assigned by the editors. Standardization work is ongoing to enhance search capabilities and cover all the relevant aspects that are not reflected in the taxonomies. For example, keywords help to distinguish case reports, single-center series, registry series, or subject reviews and to differentiate between records involving a living or deceased donor (including distinction of death determined by circulatory or by neurological criteria, where possible).

### Hosting the Library and Monitoring Its Use

The Notify Library is hosted on a dedicated website (https://www.notifylibrary.org/) where a broad range of guidance and information on vigilance and surveillance for MPHO can be found. The Library itself is open to any member of the public that can search for records or references by MPHO type or adverse occurrence type. Further tools to support vigilance have been developed over the years, including a comprehensive vigilance guidance document available for download through the related section^1^ and the possibility to access a panel of experts providing support and advice on request via the Notify website. Visits and activity on the Notify Library are monitored and analyzed using Google Analytics technology, including numbers of site visits, users, page views, bounce rates, and geographical distribution, to assess the use of the Library as a supporting tool to the vigilance and surveillance of MPHO worldwide.

## RESULTS

**FIGURE 2.**
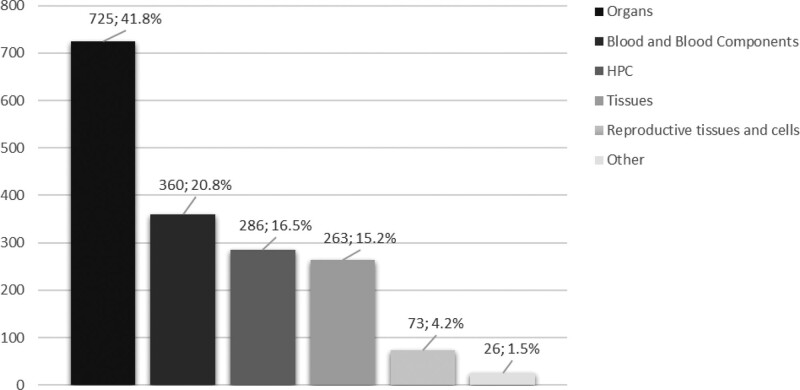
Breakdown of Notify Library records by MPHO type (as of the end of September 2020). HPC, hematopoietic progenitor cell; MPHO, medical products of human origin.

**FIGURE 3.**
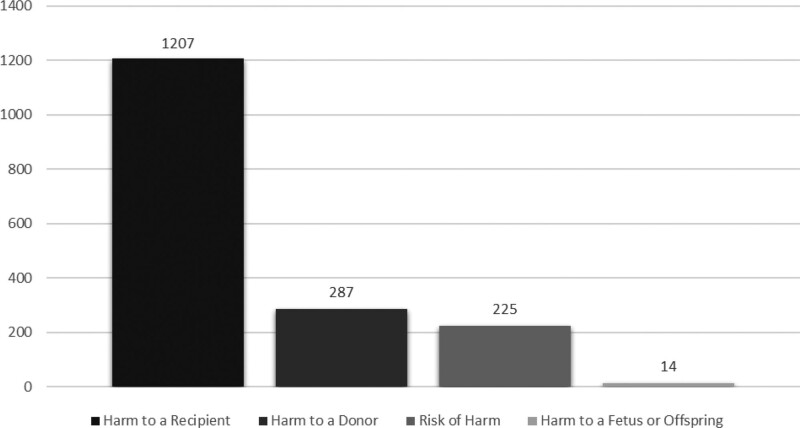
Breakdown of Notify Library records by adverse occurrence type (as of the end of September 2020).

As of the end of September 2020, the Notify Library contains 1733 instructive records and 2632 references. Analyzing the database content by MPHO type, 725 (41.8%) records are related to organs, 360 (20.8%) to blood and blood components, 286 (16.5%) to HPCs (127 collected by apheresis, 129 from bone marrow, 24 from cord blood, 6 source not specified), 263 (15.2%) to tissues, 73 (4.2%) to reproductive tissues and cells, and 26 (1.5%) to other (Figure 2). Of the 1733 records, 1207 (69.5%) were categorized as “harm to a recipient,” 287 (16.5%) as “harm to a donor,” 225 (13%) as “risk of harm,” and the remaining 14 (1%) were categorized as “harm to a fetus or offspring” (Figure 3).

### Harm to a Recipient

Half of the 1207 records classified under “harm to a recipient” were cases of infection transmission, involving organs (324/604), tissues (124/604), blood (108/604), HPCs (35/604), reproductive tissues and cells (9/604), and other (4/604). New pathogens have been added in the adverse occurrence type taxonomy in recent years: hepatitis A virus (first reported transmission of hepatitis A virus through multivisceral transplantation^12^), *Talaromyces* (first reported case of presumptive donor-derived *Talaromyces marneffei* infection through bilateral lung transplantation^13^), Borna virus (first report of donor-derived Borna disease virus 1 through kidneys and liver transplantation^14^), and Japanese Encephalitis virus (first reported case of transfusion-transmitted Japanese Encephalitis virus^15^). Cases of malignancy transmission included in the database account for 20.5% (247/1207) of the records, followed by immunological complications (205/1207; eg, ABO detrimental immunization or rejection in solid organ transplantation, acute or delayed hemolytic reactions after blood transfusion), miscellaneous complications (111/1207; eg, graft failure, dimethyl sulfoxide toxicity, undue exposure to risk or intervention, transfusion-associated circulatory overload), and noninfectious/nonmalignant transmissions (40/1207; eg, transmission of hypersensitivity or allergy by solid organ transplantation, transmission of alopecia areata or autoimmune thyroid disease following allogeneic bone marrow transplantation). HLA-related events have not been included at this time. An overview of the Notify records classified under “harm to a recipient” is available in Table 3.

**TABLE 3.** Adverse occurrence records involving harm to recipient in the Notify Library by type of MPHO (as of the end of September 2020)

**Harm to a recipient**	**Organs**	**Tissues**	**Blood and blood components**	**Cells**	**Reproductive tissues and cells**	**Other**	**Total**
Infection transmission	324	124	108	35^*a*^	9	4	604
Malignancy transmission	223	3	0	21	0	0	247
Immunological complications	20	7	153	14^*b*^	2	9	205
Miscellaneous complications	3	20	54	33^*c*^	0	1	111
Noninfectious, nonmalignant transmission	12	0	6	22	0	0	40
Total	582	154	125	125	11	14	1207

Source: Notify Library.

^*a*^Thirty-four HPCs; 1 pancreatic Islets.

^*b*^Thirteen HPCs; 1 dendritic cells.

^*c*^Thirty-one HPCs; 1 T lymphocyte; 1 olfactory mucosal cell.

HPC, hematopoietic progenitor cell.

### Harm to a Donor

There are currently 287 records in the areas of HPCs, organ, blood, musculoskeletal tissue, and oocyte donation (142, 90, 32, 18, and 5 records, respectively). Almost half of the 142 records describing HPC donation complications (94 related to peripheral blood stem cells and 48 to bone marrow) are granulocyte colony-stimulating factor related, whereas 49 of the 90 records of organ donation complications are related to surgical site complications (eg, intraoperative injury, perforation, bleeding) and postoperative infections. The 18 musculoskeletal tissue-related records concern bone and tendon autografts and refer to postretrieval donor site morbidity. Five cases of living uterus donation complications have been added to the database recently, including surgically repaired dehiscence of the vaginal cuff, temporary gluteal cramping with mobilization, and minor depression possibly due to the hormonal changes related to the simultaneous salpingo-oophorectomy performed for that patient. An overview of the Notify records classified under “harm to a donor” is available in Table 4.

**TABLE 4.** Adverse occurrence records involving harm to a donor in the Notify Library (as of end of September 2020)

**Harm to a donor**	**Organs**	**Tissues**	**Blood and blood components**	**Cells**	**Reproductive tissues and cells**	**Total**
Allergic reactions			3	1		4
Drug-related reactions				48	2	50
Embolic complications	6		2	7		15
Excessive collection/removal			1			1
Infection	13	3		10	2	28
Malignancy				3^*a*^		3
Miscellaneous complications	69	15	12	51	1	148
Toxicity			2	2		4
Vasovagal reactions			11	9		20
Other	2		1	11		14
Total	90	18	32	142	5	287

Source: Notify Library

^*a*^Two HPCs; 1 T lymphocyte.

HPC, hematopoietic progenitor cell.

### Risk of Harm

Adverse occurrences classified under “risk of harm” are described in 225 records: 104 records of loss of a suitable MPHO (46.2%; eg, due to inappropriate storage, packaging or shipment conditions, processing error, allograft damage during procurement); 73 records of unsuitable MPHO released for clinical use (32.4%; eg, retransplantation after removal of organ from donor with undetected neoplasm, bacterial contamination of infused HPC products, inadequate quality of a cornea graft or wrong size of a pulmonary heart valve discovered at the hospital, and resulting in delay in patient treatment); 25 records provide important information on where transplants proceeded from donors with transmissible diseases but no transmission occurred (11.1%; eg, renal cell carcinoma detected at procurement and excised before transplant, use of organs from known *Trypanosoma cruzi*-positive donors with or without prophylactic treatment to the recipients); and 23 records describe other adverse occurrences (10.3%; eg, gamete or embryo mix-up, barcode error leading to sample misidentification during blood grouping). An overview of the Notify records classified under “risk of harm” is available in Table 5.

**TABLE 5.** Adverse occurrence records involving a risk of harm in the Notify Library (as of the end of September 2020)

**Risk of harm**	**Organs**	**Tissues**	**Blood and blood components**	**Cells**	**Reproductive tissues and cells**	**Other**	**Total**
Donor disease without transmission	23	1		1			25
Loss of a suitable MPHO	19	33	1	21^*a*^	32		104
Mix-up	1			1	6		8
Unsuitable MPHO released for clinical use—no harm	6	52	3	3	5	4	73
Other	4	5	3	3^*b*^			15
Total	53	91	7	5	43	4	225

Source: Notify Library.

^*a*^Ninteen HPCs: 2 chondrocyte.

^*b*^Two HPCs: 1 mesenchymal stem cell.

HPC, hematopoietic progenitor cell; MPHO, medical products of human origin.

## DISCUSSION

Over the years, the role of the Notify Library as a compendium of instructive information has become increasingly clear. It provides open access to reliable, empiric evidence, and real-life experience that can be used for guideline development by professionals and health authorities when managing or investigating a specific adverse occurrence raised in the transfusion, transplantation, or assisted reproduction process or when estimating risk (eg, whether to accept organ for transplantation from a donor that poses a potential disease transmission risk). Based on cases in the Notify Library, expert reviews have been published in different fields such as orthopedics,^16^ ophthalmology,^17^ immunohematology,^18^ and transfusion medicine^19^ to raise clinician awareness of adverse reactions and events related to their practices and of how they have been detected, investigated, and managed elsewhere.

The real value of the Library, compared with a search and consultation of published articles or vigilance system reports, comes from 3 unique strengths. The first is the aggregation of reliable information in a structured and searchable format that facilitates easy access to anyone that needs it. The second is the contribution of the Notify editorial group members who share their knowledge and experience when objectively reviewing cases and building records. They provide an invaluable service to the international community by evaluating what can be learned from the cases before publication as records in the Library. The third is the large and active vigilance network that has been built and continues to grow through professional interaction and project dissemination activities worldwide. The network can be used, going forward, to support developing vigilance activities in countries and regions where these are still at an early stage and to reinforce collaboration among consolidated MPHO vigilance programs.

The value of the tool is evident from usage monitoring data. Since 2015, when an upgraded website was deployed to replace the original platform, up to December 31, 2019, 42 443 users visited the website for an overall number of 58 670 sessions, with a steady increase year by year.

Analysis of site visits by WHO region (ie, Regional Office for Africa, Eastern Mediterranean Regional Office, Regional Office for Europe, Pan American Health Organization, South-East Asia Regional Office, Western Pacific Regional Office) over the last 3 years (2017–2019) reveals one of the current limitations of the project. There are generally a significantly higher number of users from Europe and North America and considerably fewer from South America, Australia, Asia, the Middle East, and Africa. This reflects a higher number of experts who are active in the project from North America and Europe. A concerted effort to make the project more globally relevant is now a priority and is showing some success. Participation of project experts in around 15 conferences and training courses each year plays an important role in sharing information on the Library. A Notify project session held during the first congress of the African Transplantation Society in Cairo in 2019 resulted in a steady growth of visits to the site from Egypt and Iran. Similarly, when Colombia set up their MPHO vigilance system using Notify as a reference source of information, a high number of visits from that region was recorded.

An early decision not to include information published in the lay media was based on a commitment to share information that professionals could confidently rely on. Many adverse occurrences, however, are not described in published scientific articles, even though they involve important lessons for others in the field. With this limitation in mind, the project has worked to gain access to instructive, but unpublished, case information in professional or regulatory vigilance reporting programs. A partnership was built with an European Union (EU)-funded Joint Action that brought EU blood, tissue, and cell regulators together to improve inspection and vigilance approaches.^20^ The collaboration aimed at increasing the involvement of EU Member State vigilance programs in the Notify Library project. Notify provided instructions to facilitate the selection and analysis of case types with instructive value from the annual national vigilance reports submitted to the European Commission for insertion in the Library.^21^ This same approach could be replicated in the 6 WHO Regions to enhance the content of the Library with cases that are not published in international literature but are only available in national or regional vigilance programs.

Looking to the future, the project is also addressing new challenges such as horizon scanning of emerging and reemerging pathogens and their potential risk to MPHO safety. Horizon scanning activities will be a key point in future cooperation with relevant stakeholders such as WHO, the US Centers for Disease Control, and the European Centre for Disease Prevention and Control. In the framework of the Notify project, a systematic review of informal and formal reports resulting from media mining was performed in 2016 in collaboration with the department of Global Preparedness, Surveillance, and Response of WHO using the Hazard Detection and Risk Assessment System. This tool integrates a large number of electronic resources to identify common and (re)emerging infections that could constitute possible threats to MPHO safety.^22^ The experience proved valuable and led to a proposal for the establishment of a global network of public health specialists in MPHO-associated risk. The network would share expertise on horizon scanning and preparedness planning for emerging risks and could help to keep the Notify Library updated.

The importance of systematic scanning for new risks, risk identification and assessment, and structured preparation for mitigation of threats to MPHO safety at a global level is evident from the experience of the current SARS-CoV-2 health crisis, an event unprecedented in recent human history. During the pandemic, the Notify Library acted as a hub for sharing information from the international authorities and scientific societies. This included recommendations for the management of organ, tissue, and cells donation and preventive measures for the use of blood and blood components.^23^ The COVID-19 pandemic has highlighted the challenge of maintaining safe clinical application of MPHO in the context of a global crisis. The risk of not having access to MPHO treatment due to overburdening of healthcare resources and social distancing measures may prove to be one of the most adverse outcomes for patients in urgent need of MPHO, particularly organ or HPC transplantation. The project will actively explore the role of vigilance systems and expert networks in identifying new risks to MPHO safety and supply.

## ACKNOWLEDGMENTS

The Notify Library database relies on the free and voluntary contribution of outstanding experts and institutions around the world. The Italian National Transplant Centre (CNT) as WHO Collaborating Centre thanks all the experts who have contributed with their knowledge over the years to this work. The authors are most grateful to Dr Luc PJ Noel, Dr Jose Ramon Nunez, Mr Efstratios Chatzixiros, and all WHO officers who continue to support the Notify project and its database. They also express their gratitude to the Steering Group experts: Jeremy Chapman, Francis Delmonico, Elmi Muller, Alessandro Nanni Costa, Dietger Niedervieser, Phil O’Connel, Tim Pruett, Axel Rahmel, and Jaume Tort and to the invaluable review and editorial work carried out by the Notify Editorial Group Members.

Notify Editorial Group Members: Alessandra Alteri, Paul Ashford, Scott Brubaker, Mar Carmona, Mauro Costa, Jennifer DeMatteo, Dragoslav Domanovic, Beatriz Dominguez-Gil, Ted Eastlund, Carl-Ludwig Fischer-Froehlich, Lydia Foeken, Manish Gandhi, Melissa Greenwald, Paolo Grossi, Matthew Kuehnert, Oscar Len, Marilyn Levi, Kathy Loper, Marian Macsai, Jay Menitove, Kerstin Moench, Eduard Muniz, Michael Nalesnik, Paula Nolan, Wendy Paul, Mona Papari, Lizabeth Rosenbaum, Mary Townsend, Ines Ushiro-Lumb, Suzanna M. van Walraven, and Barbee Whitaker.

## Supplementary Material


