# Supplementary material for: Vigilance for Medical Products of Human Origin—Progress on the Notify Library’s Global Effort to Share Information and Learning
Source: Transplantation. 2021 Aug 19;105(9):1921-1929. doi: 10.1097/TP.0000000000003589 (PMC8376274; doi:10.1097/TP.0000000000003589)
Supplement: Supplementary file 1 [file tp-105-1921-s001.pdf]

Annex I – Taxonomy tables used to classify occurrences in a structured way.

Adverse Occurrence type taxonomy

ADVERSE OCCURRENCE TAXONOMY							
**LEVEL 1**	**LEVEL 2**	**LEVEL 3**	**LEVEL 4**	**LEVEL 1**	**LEVEL 2**	**LEVEL 3**	**LEVEL 4**
**Harm to a recipient**	**Infection**	**Viral**	Adenovirus	**Harm to a recipient**	**Infection**	**Bacterial**	Ehrlichia
			Arenavirus				Elizabethkingia
			BK virus (BKV)				Enterobacter
			Colorado tick fever virus				Enterococcus
			Cytomegalovirus (CMV)				Escherichia
			Dengue virus (DENV)				Hafnia
			Enterovirus				Klebsiella
			Epstein-Barr Virus (EBV)				Legionella
			Hepatitis A Virus (HAV)				Listeria
			Hepatitis B Virus (HBV)				Morganella
			Hepatitis C Virus (HCV)				Mycobacterium
			Hepatitis E virus (HEV)				Mycoplasma
			Human Herpes Virus 6 (HHV-6)				Oerskovia
			Human Herpes Virus 8 (HHV-8)				Orientia
			Human immunodeficiency virus (HIV)				Prevotella
			Human papillomavirus (HPV)				Propionibacterium
			Herpes simplex virus (HSV)				Proteus
			Human T-lymphotropic Virus (HTLV)				Providencia
			Influenza virus				Pseudomonas
			JC virus				Psychrobacter
			Lymphocytic choriomeningitis virus (LCMV)				Richettsia
			Parvovirus B19				Salmonella
			Rabies virus				Serratia
			Ross River virus				Staphylococcus
			Saint Louis encephalitis virus				Stenotrophomonas
			Tick-borne encephalitis virus				Streptococcus
			Zika Virus (ZIKV)				Treponema
			West Nile virus (WNV)				Veillonella
		**Bacterial**	Acinetobacter				Yersinia
			Alcaligenes			**Fungal**	Acremonium
			Anaplasma				Apophysomyces
			Bacillus				Arthrographis
			Bacteroides				Aspergillus
			Bartonella				Candida
			Brucella				Coccidioides
			Cardiobacterium				Cryptococcus
			Chlamydia				Encephalitozoon
			Citrobacter				Histoplasma
			Clostridium				Paecilomyces

ADVERSE OCCURRENCE TAXONOMY			
**LEVEL 1**	**LEVEL 2**	**LEVEL 3**	**LEVEL 4**
**Harm to a recipient**	**Infection**	**Fungal**	Rhodotorula
			Scedosporium
			Sporothrix
		**Prion**	CJD
			vCJD
		**Parasitic**	Acanthamoeba
			Babesia
			Balamuthia
			Clonorchis
			Echinococcus
			Halicephalobus
			Leishmania
			Plasmodium
			Schistosoma
			Strongyloides
			Toxoplasma
			Trypanosoma
			Wuchereria
		**Type not specified**	
	**Malignancy**	**Carcinoma of unknown primary site**	
		**Cancer, multiple types**	
		**Adrenal**	Adrenal cancer, other or type not specified
			Adrenal cortical carcinoma
			Pheochromocytoma
		**Blood and lymphoid**	Essential thrombocythemia (thrombocytosis)
			Histiocytic neoplasm, not further specified
			Hodgkin lymphoma
			Leukemia, lymphocytic, acute lymphoblastic
			Leukemia, lymphocytic, chronic
			Leukemia, lymphocytic, not further specified
			Leukemia, monocytic, acute
			Leukemia, myeloid, acute myelogenous (includes myeloid sarcoma)
			Leukemia, myeloid, chronic myelogenous
			Leukemia, myeloid, not further specified
			Leukemia, myeloid, promyelocytic
			Leukemia, type not specified
			Lymphoma, B cell, follicular
			Lymphoma, B-cell, Burkitt
			Lymphoma, B-cell, diffuse large type
			Lymphoma, B-cell, mantle zone
**Harm to a recipient**	**Malignancy**	Blood and lymphoid	Lymphoma, B-cell, marginal zone
			Lymphoma, B-cell, other or not further specified
			Lymphoma, NK cell
			Lymphoma, T cell
			Lymphoma, type not specified
			MGUS (monoclonal gammopathy of unknown significance)
			Multiple myeloma/plasmacytoma leukemia
			Myelodysplastic syndrome
			Plasmacytoma, extra medullary
			Polycythemia vera
		**Bone and cartilage**	Bone cancer, other or type not specified
			Chondrosarcoma
			Osteosarcoma
		**Breast**	Ductal adenocarcinoma
			Lobular adenocarcinoma
			Breast cancer, other or type not specified
		**Cardiovascular**	Atrial myxoma
			Cardiac, other or type not specified
		**Central nervous system**	Astrocytoma (WHO grade 1)
			Astrocytoma (WHO grade 2)
			Astrocytoma (WHO grade 3)
			Astrocytoma/glioblastoma multiforme (WHO grade 4)
			Astrocytomas and glioblastomas not further specified (WHO grade 1-4)
			CNS lymphoma
			CNS tumor, other or type not specified
			Ependymoma (WHO grade 2-3)
			Medulloblastoma (WHO grade 4)
		**Gastrointestinal**	Anal adenocarcinoma
			Anal squamous cell carcinoma
			Anus cancer, other or type not specified
			Appendix adenocarcinoma NOS
			Appendix mucinous adenocarcinoma
			Appendix cancer, other or type not specified
			Esophageal adenocarcinoma
			Esophageal squamous cell carcinoma
			Esophageal cancer, other or type not specified
			Gastric adenocarcinoma
			Gastric cancer, other or type not specified
			Gastrointestinal carcinoid/neuroendocrine tumor
			Gastrointestinal stromal tumor (GIST)

ADVERSE OCCURRENCE TAXONOMY

LEVEL 1	LEVEL 2	LEVEL 3	LEVEL 4
**harm to a recipient**	**malignancy**	**Gastrointestinal**	Large bowel adenocarcinoma
			Large bowel cancer, other or type not specified
			Small bowel adenocarcinoma
			Small bowel gastrointestinal stromal tumor (GIST)
			Small bowel cancer, other or type not specified
		**Germ cell, sex cord and related tumors**	Choriocarcinoma
			Embryonal cell carcinoma
			Granulosa cell tumor
			Leydig cell tumor
			Mixed germ cell tumor
			Seminoma/dysgerminoma
			Sertoli-Leydig cell tumor
			Teratoma
			Yolk sac tumor
			Germ cell tumor, other or type not specified
			Sex cord tumor, other or type not specified
		**Head and neck**	Nasopharyngeal carcinoma
			Ocular melanoma
			Salivary gland carcinoma
			Squamous cell carcinoma, larynx and hypopharynx
			Squamous cell carcinoma, tongue and oral cavity
			Head and neck cancer, other or type not specified
		**Kidney and urinary tract**	Angiomyolipoma
			Renal cell carcinoma
			Renal oncocytoma
			Renal cancer, other or type not specified
			Urothelial (transitional) cell carcinoma
			Bladder adenocarcinoma
			Urinary tract neoplasm, other or type not specified
		**Liver, gallbladder and bile ducts**	Cholangiocarcinoma
			Hepatocellular adenoma
			Hepatocellular carcinoma
			Liver, angiosarcoma
			Liver, hemangioendothelioma
			Liver cancer, other or type not specified
			Bile duct cancer (extrahepatic)
			Gallbladder cancer
		**Lung and lower respiratory system**	Lung cancer, adenocarcinoma
			Lung cancer, carcinoid
			Lung cancer, neuroendocrine and large cell cancer

LEVEL 1	LEVEL 2	LEVEL 3	LEVEL 4
Harm to a recipient	Malignancy	**Lung and lower respiratory system**	Lung cancer, small cell
			Lung cancer, squamous cell (bronchogenic)
			Lung cancer, other or type not specified
			Tracheal cancer
		**Neuroendocrine tumors**	Neuroendocrine carcinoma
			Neuroendocrine tumor
			Small cell carcinoma
		**Ovaries and fallopian tubes**	Endometrioid ovarian carcinoma
			Mucinous ovarian carcinoma
			Serous ovarian carcinoma
			Brenner tumor/transitional cell carcinoma
			Ovaries and fallopian tubes, other or type not specified
		**Pancreas**	Acinic/acinar cell carcinoma
			Islet cell tumor
			Pancreatic (ductal) adenocarcinoma
			Pancreatic intraepithelial neoplasia (PanIN)
			Pancreas cancer, other or type not specified
		**Parathyroid**	Parathyroid carcinoma
			Parathyroid cancer, other or type not specified
		**Pleura and peritoneum**	Mesothelioma
			Pleura and peritoneum, other or type not specified
		**Prostate**	Prostate adenocarcinoma/carcinoma
			Prostate cancer, other or type not specified
		**Skin**	Basal cell carcinoma
			Melanoma
			Merkel cell carcinoma
			Squamous cell carcinoma
			Skin cancer, other or type not specified
		**Soft tissue/sarcoma**	Angiomyolipoma
			Angiosarcoma
			Chondrosarcoma
			Epithelioid hemangioendothelioma
			Ewing's sarcoma
			Fibromatosis/desmoid tumor
			Fibrosarcoma
			Kaposi's sarcoma
			Leiomyosarcoma
			Liposarcoma
			Nerve sheath tumor NOS
			Neurilemmoma/schwannoma

ADVERSE OCCURRENCE TAXONOMY

LEVEL 1	LEVEL 2	LEVEL 3	LEVEL 4	LEVEL 1	LEVEL 2	LEVEL 3	LEVEL 4
**harm to a recipient**	**malignancy**	**Soft tissue/sarcoma**	Neurofibroma	harm to a recipient	**Miscellaneous complications**	Acute Hemolytic Reaction - non-immune	
			Osteosarcoma			Cardiovascular complications	
			Paraganglioma			Catheter related complications	
			Rhabdomyosarcoma			Delayed engraftment	
			Solitary fibrous tumor			Delayed Hemolytic Reaction - non-immune	
			Sarcoma, other or type not specified			Febrile Reaction	
		**Thyroid**	Follicular carcinoma			Graft failure	
			Medullary carcinoma			Hemosiderosis	
			Papillary carcinoma			Hypertensive Reaction	
			Thyroid cancer, other or type not specified			Hypotensive Reaction	
		**Uterus, cervix and vagina**	Endometrial carcinoma			Inappropriate clinical application	Insufficient MPHO use
			Endometrial stromal sarcoma				Eccessive MPHO use
			Cervical adenocarcinoma			Neurological complications	
			Cervical squamous cell carcinoma			Pulmonary complications	
			Cervical cancer, other or type not specified			Surgical site complications	
			Uterine cancer, other or type not specified			TACO	
	**Non-infectious, Non-malignant transmissions**	Alloimmune				TAD	
		Autoimmune				Toxicity	Citrate
		Genetic					Potassium (hyperkalemia)
		Hypersensitivity/allergy					DMSO
	**Immunological complications**	Acute Hemolytic Reaction					Ethlene oxide
		Allergic Reaction				Undue exposure to risk/intervention	
		Delayed Hemolytic Reaction				Other	
		Delayed Serologic Reaction		**harm to a donor**	**Allergic reaction**	Local	
		Detrimental immunization (MPHO other than blood)	ABO immunisation			Systemic /anaphylactic	
			Rh immunisation		**Drug related reactions**	GCSF-related	
			HLA immunisation			Ovarian Hyperstimulation Syndrome	
			Other detrimental immunization		**Embolic Complications**	Air embolism	
		Graft versus Host Disease				Fat embolism	
		Post Transfusion Purpura (PTP)				Thromboembolism	
		Passive alloimmune thrombocytopenia			**Excessive collection/removal**		
		Rejection			**Infection**		
		TRALI			**Malignancy**		

ADVERSE OCCURRENCE TAXONOMY

LEVEL 1	LEVEL 2	LEVEL 3	LEVEL 4
**Harm to a donor**	**Miscellaneous complications**	Anesthetic agents	
		Cardiovascular	
		Catheterization/Intubation	
		Gastrointestinal	
		Immunological	
		Insertion of needle	
		Metabolic	
		Neurological	
		Psychological	
		Pulmonary	
		Surgical site	
	**Procurement outside legal framework**		
	**Toxicity**		
	**Undue exposure to risk/intervention**		
	**Vasovagal Reactions**		
	**Other**		

LEVEL 1	LEVEL 2	LEVEL 3	LEVEL 4
**Harm to a fetus or offspring**	**Genetic**		
**Risk of harm**	**Loss**	Highly matched or autologous MPHO	
		Suitable organ(s)	
		Large quantity of unmatched MPHO	
	**Mix-up**	Gamete	
		Embryo	
		Incorrect MPHO applied - no harm	
	**Unsuitable MPHO released for clinical use - no harm**		
	**Other**		

MPHO type taxonomy

MPHO (Medical Products of Human Origin) TAXONOMY

LEVEL 1	LEVEL 2	LEVEL 3	LEVEL 4
**MPHO**	**Organs**	Liver	
		Heart	
		Kidney	
		Lung	
		Pancreas	
		Small bowel	
		Combined	Heart lung
			Kidney pancreas
			Liver Kidney
			Multivisceral
		Composite tissue grafts	Face
			Hand
			Uterus
		Type not specified	
	**Tissues**	Musculoskeletal	Bone
			Cartilage
			Meniscus
			Osteochondral
			Tendon or ligament
		Cardiovascular	Blood vessels
			Conduit
			Heart valves
			Pericardium
		Ocular	Conjunctiva
			Cornea
			Limbal tissue
			Sclera
		Adipose tissue	
		Amniotic membrane	
		Other fetal membranes	
		Dura mater	
		Larynx	
		Nerve	
		Parathyroiid glands	
		Placenta	
		Skin	
		Trachea	
		Umbilical cord tissue	

LEVEL 1	LEVEL 2	LEVEL 3	LEVEL 4
**MPHO**	**Cells**	Adipocytes	
		Chondrocytes	
		Dendritic cells	
		Fibroblasts	
		Genetically modified cells	
		Hepatocytes	
		HPC (hematopoietic progenitor cell)	Marrow
			Apheresis
			Cord blood
			Type not specified
		Keratinoctyes	
		Leukocytes	
		Limbal cells	
		Mesenchymal stem cells	
		Olfactory mucosal cells	
		Pancreatic Islets	
		T-lymphocytes	
	**Blood**	Whole blood	
		Red blood cells	
		Platelets	
		Plasma	
		Cryoprecipitate	
		Granulocytes	
		Type not specified	
	**Reproductive**	Embryo	
		Oocyte	
		Ovarian tissue	
		Testicular tissue	
		Sperm	
		Combined	
	**Other**	Milk	
		Fecal microbiota	
		Topical products of human origin	
	**MPHO-derived medicinal products**	Plasma derivates	
		Cell derived medicinal products	
		Tissue derived medicinal products	
		Tissue and cell derived medicinal products	
